# A time-saving method for sealing Purdue Improved Crop Storage (PICS) bags

**DOI:** 10.1016/j.jspr.2018.04.002

**Published:** 2018-06

**Authors:** Kabita Kharel, Linda J. Mason, Scott B. Williams, Larry L. Murdock, Ibrahim B. Baoua, Dieudonne Baributsa

**Affiliations:** a Department of Entomology, Purdue University, West Lafayette, IN, USA; b Spensa Technologies, West Lafayette, IN, USA; c Université de Maradi, Maradi, Niger

**Keywords:** PICS bags, Hermetic storage, *Sitophilus zeamais*, Maize storage, Bag sealing

## Abstract

Purdue Improved Crop Storage (PICS) bags were designed to reduce grain storage losses on smallholder farms. The bag consists of three layers: two high-density polyethylene liners fitted inside a woven polypropylene bag. Recently, farmer groups, development relief programs, and government food security agencies have shown interest in PICS bags for large-scale use. PICS bags are conventionally closed by a twist-tie (TT) method, which involves twisting, folding, and tying the lip of each layer individually with a cord. This is not only time and labor intensive, but also may affect the integrity of the liners. We evaluated three new bag closure methods: i) inner liner rolled onto itself and middle liner fold-tied (IR), ii) both liners folded together and tied (FT), and iii) both liners folded and tied separately (FS), along with the conventional twist tie (TT) method. The time to close partially or fully filled 50 kg-capacity PICS bags filled with maize grain was assessed. Results showed that FT was the most time-saving method, reducing bag sealing time by >34% versus the usual TT method. The average internal oxygen levels reached <2% within a week in bags containing grain highly infested with *Sitophilus zeamais*, while it remained >5% levels for less-infested bags. In both cases, insect population growth was suppressed. Oxygen depletion rates among tying methods remained the same regardless of the closure method used. When large numbers of bags need to be closed, the time-saving FT method is a good alternative PICS sealing method over the conventional twist-tie approach.

## Highlights

• Fold-tie method (FT) is an alternative method to the conventional twist-tie (TT) for sealing hermetic PICS bags.
• FT reduces the bag sealing time by >34% over TT method.
• FT can maintain the hermetic conditions of PICS bags similar to TT method.
• FT can be used by farmers and food relief agencies storing large quantity of grain in PICS bags.

## 1 Introduction

The Purdue Improved Crop Storage (PICS) program grew out of an earlier project funded by the USAID Bean/Cowpea Collaborative Research Support Program (CRSP) in 1987 to address post-harvest losses of cowpea grain on smallholder farms in West Africa. In 2007, the PICS triple-bagging technology was promoted in ten countries in West and Central Africa (Baributsa et al., 2010; Baoua et al., 2012). The PICS bag consists of two, high-density polyethylene liners fitted inside a third woven polypropylene bag. When the bag is filled with grain and sealed, metabolic activities of living organisms inside the bag deplete the available oxygen, and the oxygen reaches low levels (e.g., less than 5% by volume) within a few days (Murdock et al., 2012). The low oxygen levels suppress the development, reproduction, and the survival of insects and pathogens (Baoua et al., 2014; Tubbs et al., 2016). The PICS bags have been evaluated and shown to be effective for storage of a wide range of crops including rice, wheat, maize, sorghum, groundnut, sunflower seeds, pigeonpea, beans, and mungbean (Jones et al., 2011; Baoua et al., 2014; Baributsa, 2014; Baributsa et al., 2015; Martin et al., 2015).

The PICS technology was disseminated to smallholder farmers in West and Central Africa since 2007; and by 2012, nearly 50% of the cowpea-stored on-farm in that region was stored using PICS bags or other hermetic containers (Moussa et al., 2014). Presently, the PICS program is active in more than 25 countries in Africa and has expanded into several countries in Asia including Nepal, India, and Afghanistan (PICS newsletter, 2015). PICS technology was developed to address postharvest grain losses on smallholder farms, but overtime it has attracted the interest of large-scale users including farmers’ groups, international development relief programs, government food security agencies, and grain traders.

PICS bags used by small-scale farmers and filled with grain have conventionally been sealed-using the twist-tie method. This involves twisting the lip of each layer individually (approximately 15 inches of plastic lips remain on the top after filling 50 kg of grain into a 50 kg capacity bag), folding the lip over, and tying with a cord. While simple, the twist-tie method requires substantial effort and is time-consuming. If not done right, it may damage the inner plastic liners. The time and effort required for the twist-tie method are one of the constraints to adoption of PICS bags among potential larger-scale users, some of which may use thousands of bags. Hence, it would be useful to find a simpler and faster alternative to the conventional twist-tie closure. In the present study, we developed and evaluated alternative methods of closing PICS bag and evaluated them by (1) estimating the average time taken to close the bags, and (2) assessing the effect of each tying method on oxygen depletion rates and grain quality.

## 2 Materials and methods

### 2.1 Alternative bag closing methods

**Fig. 1:**
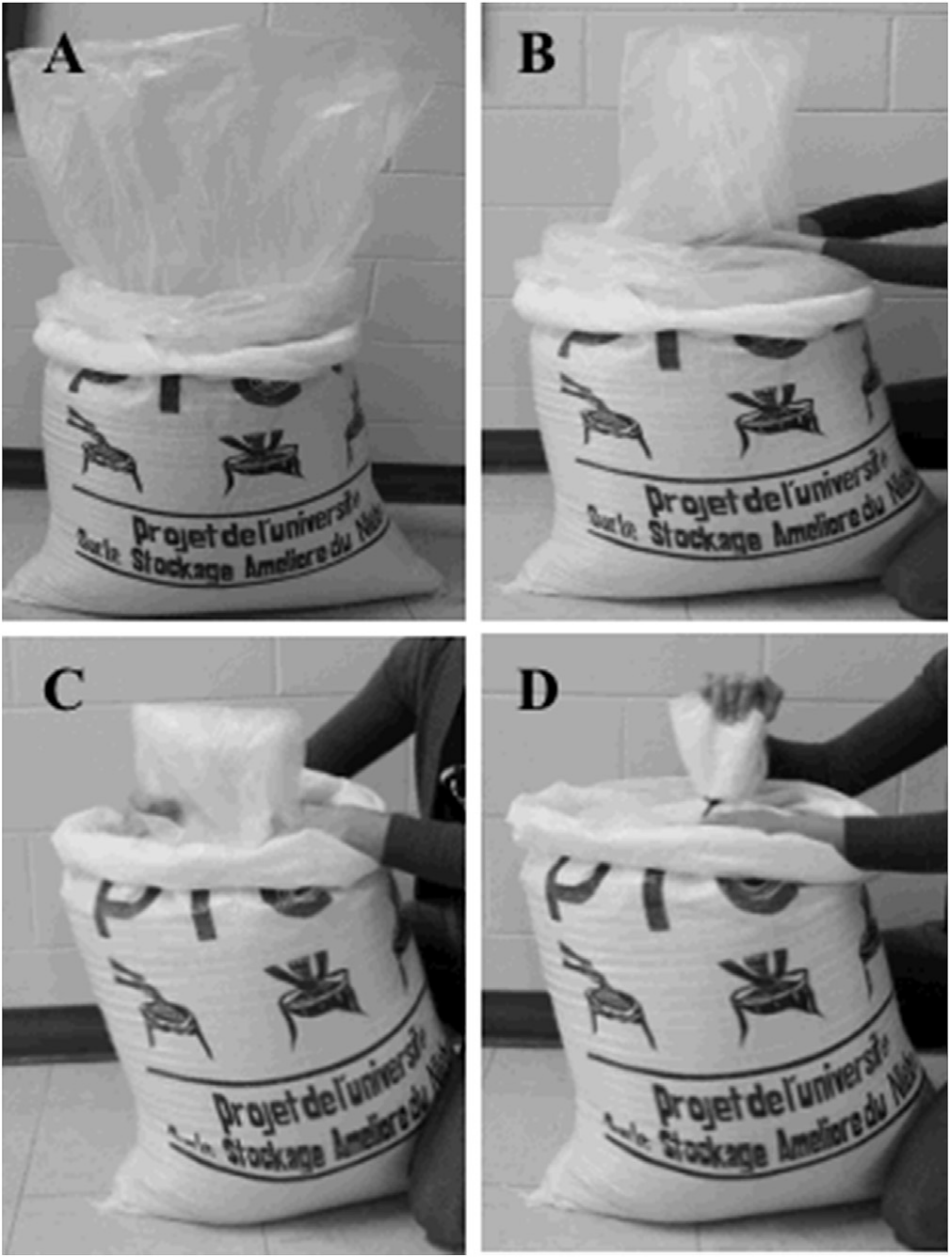
**Procedure for folding the liners of PICS bags:** A = PICS bags with grain before sealing -inner liner, middle liner folded, and the external woven bag; B = Air is being pushed out of inner plastic liner; C = the inner liner is folded down to one-half; D = the inner liner is being tied with zip-tie.

We developed three new methods of closing bags, each involved either folding without twisting or rolling the plastic liners. The closing methods were: 1) ***Inner liner Rolled (IR)*** - the inner plastic liner was rolled onto itself and the second liner folded and tied; 2) ***Folded together (FT)-*** both liners were folded together and tied; 3) ***Folded Separately (FS)***- both liners were folded and tied separately (Fig. 1). The three methods described above were compared with the conventional method of bags closure, 4) ***Twist-tied method (TT)***. In this conventional procedure currently recommended, both the inner and second plastic liners are twisted and tied separately. The twist-tie method may stress the plastic liners when the bag is used multiple times. In all of the above alternatives, the outer woven bags were twist-tied to provide firm support to the bagging system. Wear and tear on the woven bags is of less concern.

### 2.2 Experimental details

**Experiment 1:** To determine the time taken to close bags utilizing different methods, we prepared 50 kg capacity PICS bags and filled them with 35 or 50 kg maize grain. The maize variety used in the experiment was yellow maize grain (Yellow Trucker's Favorite, Lot#502) purchased from the Wax Seed Co. (Armory, Alabama). The 35 or 50 kg filled bags represent real field bag usage where farmers partially or fully fill PICS bags. Two sets of eight people were selected to seal the 35 or 50 kg bags using the four methods (TT, IR, FT, FS). The two sets of people were selected in order to increase the number of scores as the skills and abilities might vary among people. The 35 and 50 kg bags were closed during separate weeks for better handling of the experiment. The order of sealing the bags using the four methods was randomized using a random sequence generator (Haahr, 2002). The 35 kg bags were sealed by the first group of eight people using four methods every day over six days (N = 8*4*6 = 192), while the 50 kg bags were closed by the second group of eight people using four methods every day over four days (N = 8*4*4 = 128). The time taken by each person to tie the bags was recorded for each sealing method.

**Experiment 2:** To assess the effect of the four sealing methods on the performance of the PICS bags, we monitored internal oxygen levels for 90 days (d) in the bags containing maize grain artificially infested with the maize weevil, *Sitophilus zeamais* Motschulsky (Coleoptera: Curculionidae), one of the most important cosmopolitan pest of stored maize.

**Preparation of infestation grain:** Infested grain was prepared by rearing a population of *S. zeamais* in eight woven polypropylene bags filled with approximately 25 kg maize grain. Four bags were prepared by placing 15 mixed-sex *S. zeamais* adults in each bag to develop low-infested grain for a period of approximately three months. The remaining woven bags (N = 4) were infested with approximately 300 adult *S. zeamais* per bag to develop high-infested grain. Use of the infested grain ensured that all developmental stages of *S. zeamais* were present in the grain. On the first day of the experiment, six samples of 335 g each were taken from each of the four low-infested bags (N = 4 × 6 = 24 samples). Similar samples were also drawn from the high-infested bags. The number of dead and live adults were counted as a measure of the baseline infestation for each group (Table 1).

**Table 1:** Live and dead adults of *Sitophilus zeamais* (Means ± SE, in 335 g samples), and percent relative damage (Rd, Means ± SE) of low and high infested maize grain after three months of storage in PICS bags closed using different sealing methods.

Sealing methods	Low infestation			High infestation		
	Live	Dead	Rd (%)	Live	Dead	Rd (%)
Initial infestation	3.25 ± 0.56 a	2.08 ± 0.39 a	–	78.71 ± 6.70 a	28.29 ± 2.01a	–
TT after 3 months	0.06 ± 0.06 b	0.67 ± 0.21 b	0.97 ± 0.36	0.00 ± 0.00 b	10.16 ± 2.21b	0.96 ± 0.39
IR after 3 months	0.17 ± 0.09 b	0.50 ± 0.19 b	1.49 ± 0.64	0.22 ± 0.10 b	11.56 ± 3.15 b	1.16 ± 0.35
FT after 3 months	0.06 ± 0.06 b	0.67 ± 0.18 b	0.69 ± 0.38	0.00 ± 0.00 b	10.50 ± 2.12 b	0.91 ± 0.24
FS after 3 months	0.06 ± 0.06 b	0.61 ± 0.20 b	1.47 ± 0.72	0.06 ± 0.06 b	13.27 ± 2.20 b	1.41 ± 0.39
ANOVA	F_4,91_ = 22.3, *P* < 0.01	F_4,91_ = 6.65, *P* < 0.01	F_3,44_ = 0.43, *P* = 0.73	F_4,91_ = 101, *P* < 0.01	F4,91 = 12.4, P < 0.01	F_3,44_ = 0.5, *P* = 0.68

Means within the same column followed by the same letter are not significantly different at P ≥ 0.05.

Sealing methods: TT = Conventional Twist-tied, IR = Inner liner rolled and outer fold-tied, FT: Both liners folded and tied together, FS: Both plastic liners folded and tied separately.

**Experiment setup**: For the low-infestation study, twelve, 50 kg capacity PICS bags were filled with about 45 kg of clean maize grain that had been kept in a freezer (−18 °C) for at least 15 days to kill any field-related infestation and contamination. Then, approximately 5 kg of low-infested maize grain was mixed thoroughly in each bag and closed using either TT, IR, FT or FS method; with each treatment replicated three times. Similarly, for the high-infestation study, each bag received 5 kg high-infested maize grain and then closed using one of the four sealing methods. The low and high-infestation studies were initiated in separate weeks for data collection convenience. Uninfested controls were filled with 50 kg of clean maize grain and closed using the four methods. The bags were stored in Purdue University's insect quarantine room for three months. The temperature and percent relative humidity (r.h.) of the room during the experimental period (90 d) were recorded every twelve hours using USB data loggers (Lascar, Erie, PA, United States).

**Monitoring of oxygen levels inside the bags:** The oxygen levels inside the PICS bags were monitored using the Oxysense 5250i^®^ oxygen reader (Oxysense, Dallas, TX) device. The Oxysense system consists of two components: fluorescent yellow Oxydots, which are placed inside the hermetic storage system, and an ultraviolet light pen which is directed onto the Oxydots from outside the container to measure the oxygen levels inside the bag. Prior to filling bags with maize grain, we attached the Oxydots to the bottom of Petri dishes and glued the Petri dishes to the inner liner of the bags. A small area of the outer woven bag was cut away so that the Oxydots were visible through the inner liners for reading. We placed two Oxydots in each bag, one at the front side at about one-third the height of the bag; another was placed at about the two third level of the bag on the opposite side of the bag. The oxygen content in all PICS bags (infested and uninfested controls) was measured daily during the first week, twice a week over the next five weeks, and once a week thereafter. The mean oxygen level taken from the two different Oxydots was recorded as the internal oxygen content of the bags. The internal temperature and r.h. of the infested and control bags were recorded every twelve hours for 90 d by placing USB data logger inside each bag.

**Bag opening and data collection:** The bags were opened after three months of storage, and the following was assessed: i) *Insect population:* for the assessment of *S. zeamais* population development during storage, six samples of 335 g each were taken from each of the three replicates-two from the top, two from the middle and two from the bottom. The number of live or dead adults was counted for each sample; ii) *Relative grain damage:* grain damage was assessed for each bag using the method described by Alonso-Amelot and Avila-Núñez (2011). Four samples of approximately 30 g of maize grain (entirely filling small plastic containers: Spring pack slime containers 2 oz.) were taken from each bag using probes. The probe was made of 76 cm PVC pipe with inner diameters of 2.5 cm. It had five slots measuring 7.6 cm × 1.5 cm at 3.8 cm apart and could take samples from the entire depth of a grain stored in the bag (Martin et al., 2015). The grain with insect-related damage and the whole kernel were separated and counted. Then each sample was dried to 0% moisture by heating in an oven at 60 °C for five days (Williams et al., 2017). Then, the dry weight of the damaged and whole kernel for each sample was measured. The relative damage percentage was calculated using following formula (Alonso-Amelot and Avila-Núñez, 2011).

$$\text{Relative}\;\text{damage}\;\left(\text{Rd}\right)=\left[\frac{\left(Wu*Nd\right)-\left(Wd*Nu\right)}{Wu*\left(Nu+Nd\right)}\right]*100$$

Where.

Nd = Number of damaged grains,
Nu = Number undamaged grains,
Wu = Dry weight of undamaged grains,
Wd = Dry weight of damaged grains

The relative damage data were standardiszed using the baseline infestation in the control bags. Corrected relative damage was calculated using the Schneider-Orelli formula with slight modifications (r). The formula is as follows.

$$\text{Corrected}\;\text{Rd}=\frac{Infested\;samples\;Rd–Baseline\;Rd}{100-Baseline\;Rd}*100$$

iii) *Germination*: the germination test was conducted for the maize grain stored in PICS bags for 90 d. Two samples, 50 each, of undamaged grains were taken from each bag. Each set of samples were immersed in a 5% bleach solution for two minutes and washed with clean water. Then each set of seeds was wrapped in wet paper towels and placed inside small plastic containers. The plastic containers were stored in a dark location for one week in a room set at 26 ± 2 °C, 40% r.h., after which the seed samples were scored for germination. The grain was recorded as germinated if at least a part of the radical was observed breaking through the shell.

### 2.3 Statistical analyses

Statistical analyses were conducted with the General Linear Models Procedure (GLM) of the Statistical Analysis System (SAS Institute Inc. 2013, SAS Institute, Cary, NC) (SAS, 2013). The data for the time taken to seal the bags were subjected to two-way Analysis of Variance (ANOVA) to determine the significance of grain filling size (35 or 50 kg) and sealing methods (TT, IR, FT, FS). The average internal oxygen levels among the bags were compared between the degree of bag fill, sealing methods and treatment (infested and control) using three-way ANOVA. The data for the number of live and dead adults before and during the experiments were subjected to one-way ANOVA to compare the effects of sealing methods on population development. The relative grain damage (Rd) data were subjected to two-way ANOVA to measure the main effects of infestation levels (low or high) and bag sealing methods. The grain germination count data were converted to percentage values, which were transformed to angular values (Zar, 2010) before subjecting the data to one-way ANOVA to compare germination rate among sealing methods within low or high infestation study. The means between sealing methods were separated using Tukey's HSD (Honestly Significant Difference) procedure. Differences among means were considered significant at α = 0.05. The temperature and r.h. recorded through data loggers kept within PICS bags were compared against ambient temperature and r.h. using Pearson's correlation.

## 3 Results

### 3.1 Bag closing time

The two-way ANOVA showed that the average time to close the bag was significantly affected by the quantity of grain in the bags-partially (35 kg) or fully-filled (50 kg) bags (F_1, 312_ = 17.42, *P* < 0.01) and bag sealing methods (F_3, 312_ = 96.92, *P* < 0.01). There was no interaction between the quantity of grain in the bag and sealing methods (F_3, 312_ = 0.99, *P* = 3.976). Subsequent one-way ANOVA for each sealing method between 35 kg and 50 kg bag showed that the sealing time was significantly different only for FT (F_1, 78_ = 7.68, *P* < 0.01) and FS (F_1, 78_ = 8.99, *P* < 0.01).

Additionally, the bag closing time was significantly different among different sealing methods for both 35 kg (F_3, 188_ = 54.80, *P* < 0.01) and 50 kg (F_3, 124_ = 45.54, *P* < 0.01) bags. For the 35 kg and 50 kg filled bags, the average bag sealing times were- FT: 51.55 and 46.78 sec, respectively; IR: 61.95 and 57.87 sec, respectively; TT: 76.91 and 72.5 sec, respectively; and FS: 83.31 and 73.25 sec, respectively. When the data for 35 and 50 kg bags were combined, the time for closing the bags was significantly different for sealing methods (F_3, 316_ = 92.14, *P* < 0.01). The most time-efficient method in descending order was FT (49.73 sec), IR (60.32 sec), TT (75.15 sec), and FS (79.28 sec) (Table 2).

**Table 2:** Time required to seal PICS bags using the four different sealing methods.

Sealing methods	Bag closing time (sec) (Mean ± SE)		
	35 kg	50 kg	Combined data
TT	76.91 ± 2.30 a	72.50 ± 2.22 a	75.15 ± 1.65 a
IR	61.95 ± 1.70 b	57.87 ± 1.64 b	60.32 ± 1.23 b
FT	51.55 ± 1.19 c	46.78 ± 1.30 c	49.73 ± 0.92 c
FS	83.31 ± 2.31 a	73.25 ± 2.20 a	79.28 ± 1.73 a
ANOVA	F_3,188_ = 54.80, *P* < 0.01	F_3,124_ = 45.54, *P* < 0.01	F_3,316_ = 92.14, *P* < 0.01

Means within the same column followed by the same letter are not significantly different at P ≥ 0.05.

Sealing methods: TT = Conventional Twist-tied, IR = Inner liner rolled and outer fold-tied, FT: Both liners folded and tied together, FS: Both plastic liners folded and tied separately.

### 3.2 Oxygen levels inside bags

**Fig. 2:**
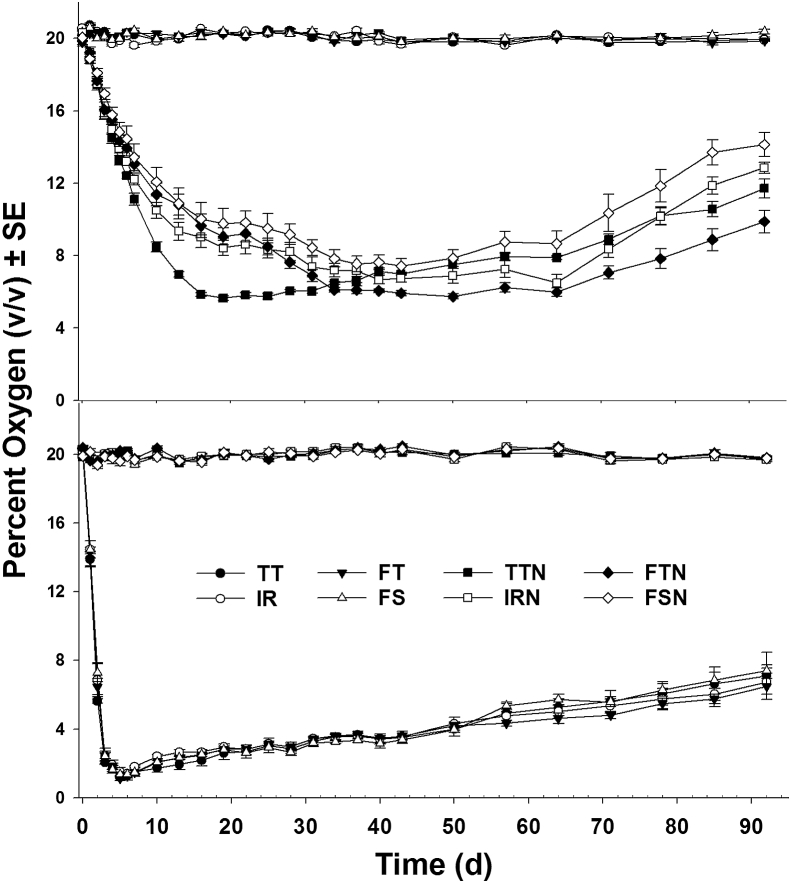
**Internal oxygen levels (v/v%) in the PICS bags sealed with different sealing methods.** Top: low-infested bags; bottom: high-infested bags. 1. Infested bags: TT = Conventional Twist-tied, IR = Inner liner rolled and outer fold-tied, FT: Both liners folded and tied together, FS: Both plastic liners folded and tied separately; 2. Control, non-infested, bags: TTN = Conventional Twist-tied, IRN = Inner liner rolled and outer fold-tied, FTN: Both liners folded and tied together, FSN: Both plastic liners folded and tied separately.

The average oxygen level inside PICS bags were significantly different between infestation levels (low or high infested bags) (F_1, 1287_ = 292.24, *P* < 0.01), and between infested grain and controls (F_1, 1287_ = 4646.22, *P* < 0.01), but not for sealing methods (TT, IR, FT, FS) (F_3, 1287_ = 1.52, *P* = 0.206). There were no interactions between infestation level and sealing method (F_3, 1287_ = 1.16, *P* = 0.324). The data showed that the average oxygen levels reached <2% within 5 d of sealing for high-infested bags, while it reached <10% levels only after 10 d of sealing for the low-infested bags- but never reached 2% (Fig. 2). Oxygen levels for controls of both high and low-infestation levels did not differ from the initial oxygen levels and were not significantly different after 90 d (F_3, 320_ = 0.65, *P* = 0.581; F_3, 320_ = 1.27, *P* = 0.283, respectively). The oxygen levels among the control bags remained between 19.61 ± 0.13 to 20.72 ± 0.13% during the entire period of study (Fig. 2).

For the low-infested treatment, the average internal oxygen level inside the PICS bags was significantly different among sealing methods (F_3, 320_ = 3.10, *P* = 0.027). The average oxygen levels for TT (9.85 ± 0.47) was not significantly different from FT (10.31 ± 0.48) and IR (10.68 ± 0.45), but significantly lower compared to FS (11.78 ± 0.44). For the high-infested bags, oxygen levels showed that there was no significant difference among the sealing methods (F_3, 320_ = 0.05, *P* = 0.986). The average oxygen levels for each sealing method in high-infested bags dropped to 1.41 ± 0.11% levels at some point during the course of the study.

### 3.3 Insect population development and relative damage to grain

The number of live and dead adults of *S. zeamais* was determined for each bag after 90 d of storage. The one-way ANOVA showed that all of the sealing methods significantly suppressed insect population development in both low and high infested bags (F_4,91_ = 22.3, *P* < 0.01 and F_4,91_ = 101, *P* < 0.01, respectively) (Table 1). For the estimates percent relative damage (Rd), no significance difference was observed between the infestation levels (high or low) and the sealing methods (Table 1). Subsequent one-way ANOVA within infestation levels showed no difference in sealing methods at low or high infestation (F_3,44_ = 0.43, *P* = 0.73 and F_3,44_ = 0.51, *P* = 0.68, respectively).

### 3.4 Maize germination

The germination of maize grain was not significantly different among treatments in low and high-infested maize (Table 3). In addition, maize germination was not affected by the sealing method within both controls and treatments for low and high-infested maize. The germination rates ranged between 70 and 95% among the sealing methods for both low and high-infested bags, thereby producing high standard errors (SE).

**Table 3:** Germination (Mean ± SE) of low and high infested maize grain after three months of storage in PICS bags closed using different sealing methods.

Sealing methods	Percent Germination			
	Low infestation study		High infestation study	
	Control	Infested	Control	Infested
Initial	82.4 ± 1.3		74.6 ± 1.1	
TT after 3 months	82.0 ± 5.9	86.3 ± 3.1	71.0 ± 5.6	76.7 ± 6.8
IR after 3 months	80.0 ± 1.9	78.3 ± 1.8	77.0 ± 3.5	77.3 ± 4.1
FT after 3 months	85.0 ± 5.4	83.7 ± 5.6	75.7 ± 3.2	74.7 ± 4.3
FS after 3 months	81.7 ± 1.9	82.3 ± 3.3	74.0 ± 4.4	75.3 ± 5.6
ANOVA	F_3,8_ = 0.59, *P* = 0.63	F_3,8_ = 1.05, *P* = 0.42	F_3,8_ = 0.46, *P* = 0.72	F_3,8_ = 0.09, *P* = 0.96

Means within the same column are not significantly different at P ≥ 0.05.

Sealing methods, Sealing methods: TT = Conventional Twist-tied, IR = Inner liner rolled and outer fold-tied, FT: Both liners folded and tied together, FS: Both plastic liners folded and tied separately.

### 3.5 Temperature, relative humidity

The internal temperatures of the bags were strongly and positively correlated with room temperature for all sealing methods. The Person's correlation value, *P* for TT, IR, FT, and FS were 0.991, 0.990, 0.991, and 0.992, respectively (data not shown). However, we found very weak correlation between r.h. for room and the sealed bags. The Person's correlation value, *P* between r.h. for room and TT, IR, FT, and FS methods were −0.099, −0.021, −0.051, and −0.055, respectively (data not shown).

## 4 Discussion

Our results showed that the FT (tying both liners together) and the IR methods (required tying only one of the plastic liners) reduced the bag sealing time by 34% and 20%, respectively. Both FS and TT methods took a bit longer time because they required tyings the two plastic liners. No significant difference was observed between FS and TT regarding the average time to close the bags. Sealing a 50 kg capacity bag filled with maize took less time compared to bags filled with only 35 kg maize for all tying methods. However, the sealing was only significantly different between the 50 kg and 35 kg bags with the FT and FS methods. There might be several reasons for the reduced time to close 50 kg maize bags compared to 35 kg bags. Since the 35 kg capacity was not filled to the top, there was a larger lip (61 cm) of the plastic liners (compared to the 50 kg capacity bag; 38 cm) that needed to be folded or twisted to close the bags and this requires more time. Additionally, the large lips required extra time to force out all the trapped air from the bags before sealing the liners. Typically, large-scale and commercial farmers in developing nations fill the PICS bags to their capacity and do not repeatedly open and close them to remove grain, unlike small-scale farmers. Therefore, both the FT and IR methods could greatly benefit the large-scale farmers and traders by reducing the time needed to seal the bags.

All of the alternative methods tested for sealing the bags maintained the low oxygen levels similar to the conventional twist-tie method over the extended storage period. We found that the average internal oxygen levels reached <2% (v/v) within 5 d of sealing for only high-infested bags. This is due to the high population of *S. zeamais* in highly-infested bags that accounted for the much faster consumption of oxygen. This extended hypoxic condition inside the bags not only killed the existing immature and adults of *S. zeamais* but also further suppressed population increase (Navarro, 2006; Baoua et al., 2014). However, we noted that after a few days of reaching the lowest oxygen levels, the oxygen readings began to rise slowly. Because triple-layer plastic liners are not perfectly impermeable to oxygen, we speculate that the atmospheric oxygen began to leak into the system after insects were dead and hence slowly raising the oxygen level inside the bag. Previous studies have documented similar trends (Jones et al., 2011; Baoua et al., 2012). Nevertheless, at this point nearly all the insects inside the bag are dead, and insect population growth has been arrested, so these small and slow increases in oxygen do not lead to increase in the numbers of insects.

No differences in relative damage were observed in grain stored in PICS bags tied with each of the four methods. We found no or at most one *S. zeamais* adult per kg sample at 90 d after storage. All four methods were effective at suppressing insect development. This may be due to the cessation of the feeding activities by *S. zeamais* when the oxygen levels have begun to drop after sealing of the bags (Murdock and Baoua, 2014). Our finding is consistent with previous studies that show PICS bags can severely restrict the flow of oxygen into the bags and reduce the insect population growth and survival (Baoua et al., 2012; Njoroge et al., 2014; Williams et al., 2017). Additionally, we observed no significant difference in relative damage to maize grain between the low and highly-infested maize bags. This may be due to a quick drop in oxygen level that reached <2% levels within 5 d for highly-infested bags; hence *S. zeamais* stopped feeding much earlier in high-infested bags compared to low-infested bags. The estimate of the relative damage is based on the dry weight of the grain. This measure not only takes into consideration how many grains were damaged, but it also considers the severity of the damage (Alonso-Amelot and Avila-Núñez, 2011). The assumed short duration of feeding in highly-infested bags might have resulted in the minimal feeding damage similar to that seen in the low-infested bags.

For all closing methods, the germination rates of maize grain stored in PICS bags was not different compared to the baseline value. This is in agreement with the previous studies (Baoua et al., 2012; Williams et al., 2016; Afzal et al., 2017) which stated that PICS bags do not compromise the germination rate of the stored seeds. The internal temperature in PICS bags had a strong positive correlation with external temperature. Additionally, the r.h. inside the bags remains stable for all closing methods throughout the study period. Williams et al. (2017) also observed stable r.h. in the PICS bags storing maize grain. The buffering effect of PICS bags against external factors, especially r.h. is beneficial, particularly in the regions where the external humidity fluctuates greatly during the year. Such changes may affect grain quality and impact seed viability.

Overall, our study provides evidence that folding and tying both liners together (FT) is effective at reducing the time to seal a PICS bag and can serve as an alternative method to the conventional twist-tie technique. The time-saving fold-tie method may attract large-scale users of PICS bags including commercial grain traders, development and government food security agencies, and thus expand the use of PICS technology into new markets. Our study further confirms that PICS bags control of *S. zeamais* while maintaining seed germination.
